# Effects of Apamin on MPP^+^-Induced Calcium Overload and Neurotoxicity by Targeting CaMKII/ERK/p65/STAT3 Signaling Pathways in Dopaminergic Neuronal Cells

**DOI:** 10.3390/ijms232315255

**Published:** 2022-12-03

**Authors:** Jihyun Park, Kyung Mi Jang, Kwan-Kyu Park

**Affiliations:** 1 Department of Pathology, College of Medicine, Catholic University of Daegu, Daegu 42472, Republic of Korea; 2 Department of Pediatrics, College of Medicine, Yeungnam University, Daegu 42415, Republic of Korea

**Keywords:** Parkinson’s disease, apamin, SK channel, oxidant stress, ROS, apoptosis

## Abstract

Parkinson’s disease (PD), a neurodegenerative disorder, is characterized by the loss of dopaminergic (DA) neurons. The pathogenesis of PD is associated with several factors including oxidative stress, inflammation, and mitochondrial dysfunction. Ca^2+^ signaling plays a vital role in neuronal signaling and altered Ca^2+^ homeostasis has been implicated in many neuronal diseases including PD. Recently, we reported that apamin (APM), a selective antagonist of the small-conductivity Ca^2+^-activated K^+^ (SK) channel, suppresses neuroinflammatory response. However, the mechanism(s) underlying the vulnerability of DA neurons were not fully understood. In this study, we investigated whether APM affected 1-methyl-4-phenyl pyridinium (MPP^+^)-mediated neurotoxicity in SH-SY5Y cells and rat embryo primary mesencephalic neurons. We found that APM decreased Ca^2+^ overload arising from MPP^+^-induced neurotoxicity response through downregulating the level of CaMKII, phosphorylation of ERK, and translocation of nuclear factor NFκB/signal transducer and activator of transcription (STAT)3. Furthermore, we showed that the correlation of MPP^+^-mediated Ca^2+^ overload and ERK/NFκB/STAT3 in the neurotoxicity responses, and dopaminergic neuronal cells loss, was verified through inhibitors. Our findings showed that APM might prevent loss of DA neurons via inhibition of Ca^2+^-overload-mediated signaling pathway and provide insights regarding the potential use of APM in treating neurodegenerative diseases.

## 1. Introduction

The pathological hallmarks of Parkinson’s disease (PD) are the progressive loss of dopaminergic neurons in the substantia nigra pars compacta (SNpc) and the widespread aggregation of alpha-synuclein (aSYN) in part of the midbrain, which is involved in motor control [1]. The neurotoxin 1-methyl-4-phenyl pyridinium (MPP^+^) is an active metabolite of 1-methyl-4-phenyl-1,2,3,6-tetrahydropyridine (MPTP) [2]. It is highly toxic to dopaminergic neurons and is used to establish various in vitro and in vivo experimental models of PD [3]. Systemically administered MPTP crosses the blood–brain barrier and is taken up by glial cells, where it is metabolized/oxidized to MPP^+^ [4]. MPP^+^ is then released and is specifically taken up by dopaminergic neurons via dopamine transporters, inhibiting the activity of mitochondrial complex I [5]. The consequences of mitochondrial dysfunction, thus induced by MPP^+^, are the inhibition of oxidative phosphorylation [6], ATP depletion [7], production of reactive oxygen species (ROS) [8], disturbance in calcium (Ca^2+^) homeostasis [9], oxidative stress [10], explosive release of proinflammatory cytokines [11], mitochondrial depolarization, and permeability transition, which lead to apoptotic death of dopaminergic neurons [6,12].

Although the etiology of PD is unclear, evidence suggests that abnormal protein aggregation, mitochondrial dysfunction, dysregulated Ca^2+^ homeostasis, and oxidative stress may be involved in neurodegeneration [13]. Therefore, preventing the degeneration of dopaminergic neurons has been identified as a possible therapeutic mechanism to prevent or treat PD [14].

Ca^2+^ is important for many cellular processes, such as proliferation, growth, differentiation, development, and cell death [15]. In recent years, it has been suggested that the changes in Ca^2+^ homeostasis play a key role in the degeneration of dopaminergic neurons [16]. For example, in mitochondria and endoplasmic reticulum (ER), changes in Ca^2+^ homeostasis affect neuronal survival, which is closely associated with PD [17]. In addition, abnormally high levels of intracellular free-Ca^2+^-induced overproduction of free radicals, such as reactive oxygen species (ROS), can activate stress cascade and related signaling pathways, resulting in apoptosis [12].

The small-conductivity Ca^2+^-activated K^+^ (SK) channels have emerged as potential tools for neuronal protection [18]. SK channel subtypes present alternative pharmacology and distribution in the nervous system [19]. They control the firing pattern of midbrain dopaminergic neurons in vivo [20], and could play an important role in modulating the apoptosis of dopaminergic neurons observed in PD [15]. Ca^2+^ overload was also confirmed when MPP^+^-induced oxidative stress, ER stress, and selective death of dopaminergic neurons occurred [12,21,22,23]. 

Apamin (APM) is a powerful and selective antagonist of the SK channel found in apitoxin [24]. We have reported the anti-inflammatory, antifibrotic, and anticancer properties of bee venom and its major components, melittin, and apamin [25,26,27,28,29]. In addition, we recently investigated and reported the effect of APM on nonneoplastic disease [30]. Bee venom and its components have also been suggested to have beneficial effects in the treatment of PD [26,31,32,33,34,35,36]. We recently reported that APM inhibits neuroinflammatory responses by regulating the interaction between SK channel/TLR4 and MAPK-EKR/NFκB/STAT signaling [37]. Therefore, APM is a promising candidate for treating neurotoxicity and has the potential to prevent and treat various neurological disorders.

Although our prior studies and those by other researchers have investigated the physiological and pharmacological functions of APM, the effects of APM on Ca^2+^ overload and oxidative stress caused by MPP^+^ in dopaminergic neurons has not been evaluated as a molecular mechanism. Therefore, in this study, we evaluated the potential therapeutic effects of APM on the signal transduction pathway involved in dopaminergic neurotoxicity.

## 2. Results

### 2.1. APM Protects SH-SY5Y Cells and Rat Embryo Primary Mesencephalic Neurons against MPP^+^-Induced Neurotoxicity 

To determine cytotoxicity, SH-SY5Y cells and rat embryo primary mesencephalic neurons treated with different concentrations of MPP^+^ (1–5 mM or 10–200 μM) and APM (0.1 to 2 μg/mL) for 24 h were analyzed by the established CCK8 assay. Treatment of SH-SY5Y cells with MPP^+^ concentrations ranging from 1 mM showed mild growth inhibitory activity with a 10 % decrease in cell viability at 2 mM and exhibited some toxicity (25%) at 3 mM (Figure S1a). In addition, cytotoxicity was 25% at 50 μM MPP^+^ in rat embryonic primary mesencephalic neurons (Figure S2a). We also confirmed these results at the cellular level (Figures S1b and S2b). The morphology of SH-SY5Y cells and rat embryonic primary mesencephalic neurons showed full cell bodies and extending neurites. After exposure to 3 mM or 50 μM MPP^+^, cells became sparsely distributed, and displayed growth inhibition and development of short neurites with few branches. This result was consistent with changes of cell viability. αSYN expression increased depending on the MPP^+^ concentration and expression of the dopaminergic neurons marker, tyrosine hydroxylase (TH), decreased (Figures S1c and S2c).

The cytotoxic effects of APM on SH-SY5Y cells and rat embryonic primary mesencephalic neurons were examined through a CCK assay before investigating its pharmacological potential. Treatment of SH-SY5Y cells and rat embryonic primary mesencephalic neurons with APM exhibited some toxicity at 0.5 μg/mL (Figures S1d and S2d). APM significantly increased the viability of 3 mM or 50 μM MPP^+^-stimulated SH-SY5Y cells and rat embryonic primary mesencephalic neurons compared to cells treated with only MPP^+^ (Figures S1e and S2e). Based on these results, the optimal APM concentration for subsequent experiments was set as 0.5 μg/mL for 3 mM or 50 μM MPP^+^-stimulated dopaminergic neurons. 

**Figure 1:**
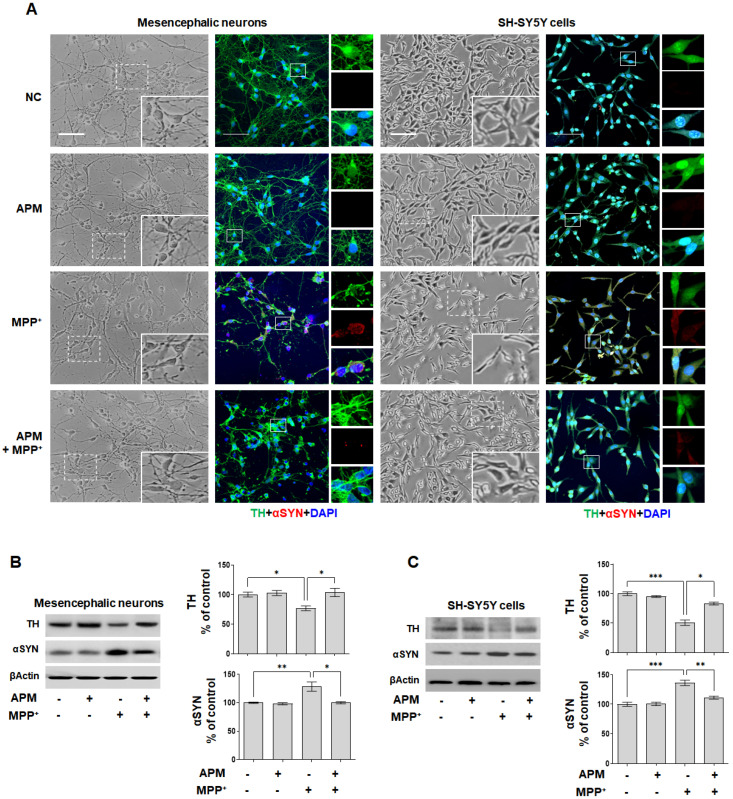
Effects of APM on MPP^+^-induced TH and αSYN expression in dopaminergic neuronal cells. SH-SY5Y cells and rat embryo primary mesencephalic neurons were incubated in the presence or absence of APM for 1 h and then treated with MPP^+^ for 24 h. (**A**) The morphological changes, TH and αSYN expression in dopaminergic neurons after exposure to MPP^+^ in the presence or absence of APM. Immunofluorescence staining for TH (green) and αSYN (red) localization. Cells were counterstained with DAPI (blue). Scale bars: 50 μm. APM strongly reduced expression of TH reduction and accumulation of αSYN in rat embryo primary mesencephalic neurons (**B**), and SH-SY5Y cells (**C**). βActin was used to confirm equal sample loading. Immunoblotting was quantified by densitometric analysis. The data are representative of three independent experiments and quantified as mean values ± SEM. Tukey’s multiple comparison test, * *p* < 0.05, ** *p* < 0.01, *** *p* < 0.001 compared to normal control.

To determine whether APM could regulate the expression of MPP^+^-induced TH and αSYN, SH-SY5Y cells and rat embryo primary mesencephalic neurons were incubated in the presence or absence of APM for 1 h and then treated with MPP^+^ for 24 h. The rat embryo primary mesencephalic neurons and SH-SY5Y cells grew well, showing obvious neurites, and the cells treated with only APM did not show any difference in cell growth compared to normal cells (Figure 1A). When rat embryo primary mesencephalic neurons and SH-SY5Y cells were exposed to MPP^+^, neurites were reduced and cell debris increased; however, they were recovered with APM co-treatment. Immunofluorescence staining with an antibody against TH and αSYN revealed that rat embryo primary mesencephalic neurons and SY-SY5Y cells were healthy TH-positive neurons with extensive neurites. However, treatment of cells with MPP^+^ for 24 h reduced the number of TH-positive cells and induced accumulation of αSYN. When the cells were treated with only APM, the number and morphology of TH-positive neurons did not change. Addition of APM to MPP^+^-treated cells seemed to protect them against the loss of TH-positive neurons and accumulation of αSYN. These results were also confirmed for SH-SY5Y cells. Consistent with immunofluorescence staining, APM attenuated TH reduction and accumulation of αSYN in rat embryo primary mesencephalic neurons (Figure 1B). These expressions were also confirmed in SH-SY5Y cells (Figure 1C). These results put together indicated that APM protected dopaminergic neurons from neurotoxicity induced by MPP^+^.

### 2.2. APM Ameliorates MPP^+^-Induced Mitochondrial-Dependent Apoptosis Pathway in SH-SY5Y and Rat Embryo Primary Mesencephalic Neurons

**Figure 2:**
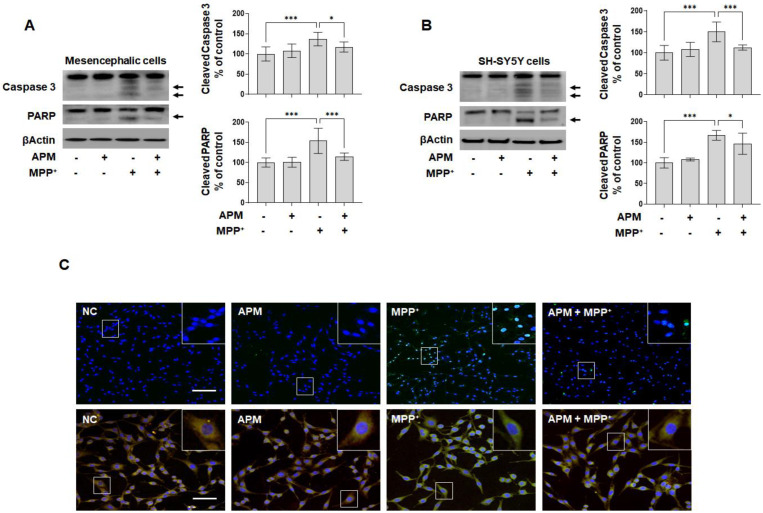

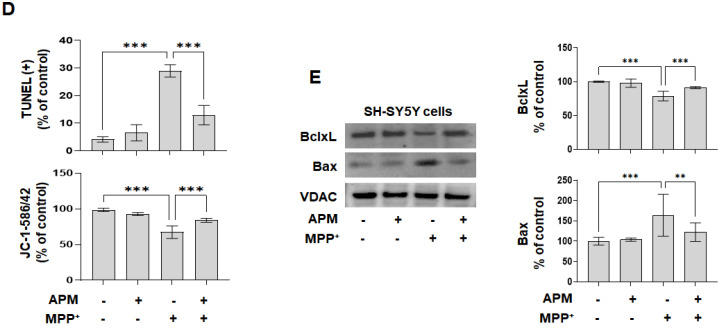
APM protects MPP^+^-induced mitochondria-dependent neurotoxicity in dopaminergic neuronal cells. SH-SY5Y cells and rat embryo primary mesencephalic neurons were incubated in the presence or absence of APM for 1 h and then treated with MPP^+^ for 12 h or 24 h. Expression of cleaved-Caspase3 and cleaved-PARP in rat embryo primary mesencephalic neurons (**A**) and SH-SY5Y cells (**B**) were detected by immunoblotting. βActin was used to confirm equal sample loading. Arrows: cleaved form. (**C**) SH-SY5Y cells were evaluated by fluorescence microscopy on the basis of morphological criteria after TUNEL staining (upper, scale bars: 100 μm) and JC-1 mitochondrial staining (lower, scale bars: 50 μm) and immunofluorescence positive cells were quantified by densitometric analysis (**D**). Nuclei were stained with DAPI. APM regulates mitochondrial apoptotic proteins (**E**). VDAC was used as mitochondrial loading control. Immunoblotting was quantified by densitometric analysis. The data are representative of three independent experiments and quantified as mean values ± SEM. Tukey’s multiple comparison test, * *p* < 0.05, ** *p* < 0.01, *** *p* < 0.001 compared to normal control.

Cell morphology and viability were identified (Figures S1 and S2) to understand the neurotoxic effect of MPP^+^ on the growth of dopaminergic neuronal cells. Since apoptosis is one of the important steps in the pathogenesis of PD, we hypothesized that APM could protect dopaminergic neuronal cells by inhibiting the apoptotic pathway. APM inhibited the cleavage of caspase-3 and PARP, which are apoptosis marker proteins, in MPP^+^-induced rat embryo primary mesencephalic neurons and SH-SY5Y cells (Figure 2A,B). Consistent with these results for APM, it was confirmed that MPP^+^ induced TUNEL positive cells in SH-SY5Y cells (Figure 2C,D). Next, we used JC-1 staining to measure the mitochondrial membrane potential to examine whether mitochondrial membrane integrity was affected by APM. SH-SY5Y cells were incubated in the presence or absence of APM for 1 h and then treated with MPP^+^ for 12 h. APM clearly recovered the number of mitochondria, which was observed as decreased membrane potential in MPP^+^-stimulated SH-SY5Y cells. Furthermore, the anti-apoptotic protein BclxL was significantly increased, while the pro-apoptotic protein Bax was significantly decreased, by APM in MPP^+^-stimulated SH-SY5Y cells (Figure 2E). Thus, these results indicated that APM could protect dopaminergic neuronal cells from mitochondrial dependent apoptosis caused by MPP^+^.

### 2.3. APM, a Strong Inhibiter of SK2 Channel, Protects MPP^+^-Induced Loss of TH-Positive Neurons and Accumulation of αSYN via Ca^2+^ Signaling

**Figure 3:**
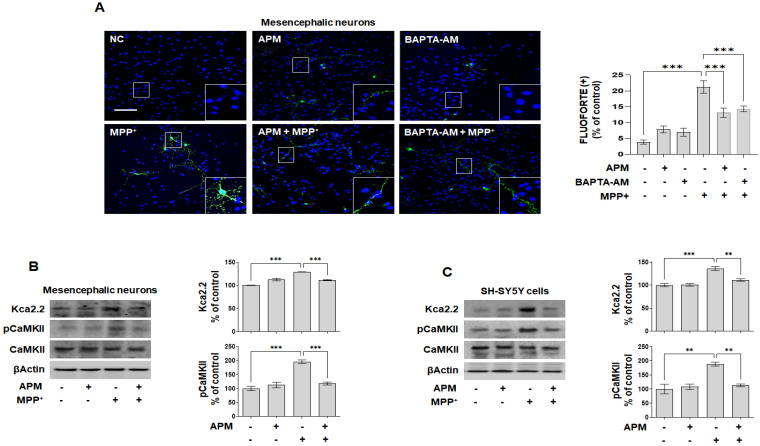
APM inhibited MPP^+^-induced SK channels in dopaminergic neuronal cells. SH-SY5Y cells and rat embryo primary mesencephalic neurons were incubated in the presence or absence of APM or BAPTA-AM (5 μM) for 1 h and then treated with MPP^+^ for 24 h. (**A**) Intracellular Ca^2+^ overload positive cells of rat embryo primary mesencephalic neurons were evaluated using fluorescence microscopy using FLUOFORTE staining (green, scale bars: 100 μm). Nuclei were stained with DAPI (blue). APM significantly inhibit MPP^+^-induced Kca2.2 and pCaMKⅡ expression in rat embryo primary mesencephalic neurons (**B**) and SH-SY5Y cells (**C**). βActin was used to confirm equal sample loading. Immunoblotting and immunofluorescence positive cells were quantified by densitometric analysis. The data are representative of three independent experiments and quantified as mean values ± SEM. Tukey’s multiple comparison test, ** *p* < 0.01, *** *p* < 0.001 compared to normal control.

APM is a specific inhibiter of SK2 channels [24], and we recently reported that APM inhibits neuroinflammatory responses through SK2 channels [37]. Ca^2+^ channels play an important role in the loss of TH-positive neurons and the accumulation of αSyn, and its inhibition is essential for the protection of dopaminergic neurons [12,16]. First, the expression of Kca2.2 was increased in a concentration-dependent manner with MPP^+^ in SH-SY5Y cells and rat embryonic primary mesencephalic neurons (Figures S1c and S2c). Next, to examine whether APM itself regulated the SK2/Kca2.2 channel and Ca^2+^ signaling, SH-SY5Y cells and rat embryo primary mesencephalic neurons were treated with APM for 1 h followed by MPP^+^ for 24 h, followed by immunoblotting and immunofluorescence staining. First, we observed intracellular localization of Ca^2+^ overload. In Figure 3A, APM and BAPTA-AM significantly inhibited FLUOFORTE positive cells by MPP^+^ in rat embryo primary mesencephalic neurons. In addition, APM inhibited the expression of Kca2.2 and pCaMKII induced by MPP^+^ in rat embryo primary mesencephalic neurons and SH-SY5Y cells (Figure 3B,C). We also confirmed that MPP^+^-induced TH reduction and αSYN expression were protected by BAPTA-AM in SH-SY5Y cells (Figure S3a). These results suggested that APM could alter MPP^+^-induced neurotoxicity in dopaminergic neuronal cells by inhibiting Ca^2+^ overload. 

### 2.4. APM Alleviates MPP^+^-Induced Neuroinflammatory Response, Oxidative and ER Stress 

**Figure 4:**
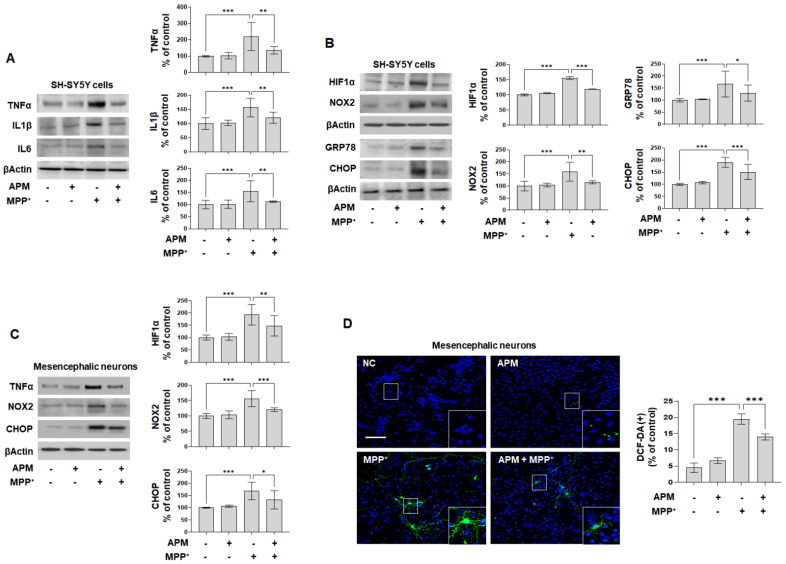
APM alleviated MPP^+^-induced neuroinflammatory response, oxidative stress, and ER stress. Dopaminergic neuronal cells were incubated in the presence or absence of APM for 1 h and then treated with MPP^+^ for 24 h. Expression of inflammatory cytokines (**A**), HIF1α, NOX2, GRP78, and CHOP (**B**) in SH-SY5Y cells were analyzed by immunoblotting. (**C**) Expression of TNFα, NOX2, and CHOP in rat embryo primary mesencephalic neurons were confirmed by immunoblotting. βActin was used to confirm equal sample loading. (**D**) ROS generation in rat embryo primary mesencephalic neurons was detected by DCF-DA staining (green, scale bars: 100 μm). Nuclei were stained with DAPI (blue). Immunoblotting and immunofluorescence positive cells were quantified by densitometric analysis. The data are representative of three independent experiments and quantified as mean values ± SEM. Tukey’s multiple comparison test, * *p* < 0.05, ** *p* < 0.01, *** *p* < 0.001 compared to normal control.

MPP^+^ causes mitochondrial dysfunction, neuroinflammation, oxidative stress, and ER stress [38]. To evaluate the impact of APM on MPP^+^-mediated neuroinflammatory response, SH-SY5Y and rat embryo primary mesencephalic neurons were treated with APM for 1 h followed by MPP^+^ for 24 h, immunoblotting, real-time PCR, ELISA, and immunofluorescence staining. Increased TNFα, IL1β, and IL6 expression was significantly inhibited in MPP^+^-stimulated SH-SY5Y cells by APM administration (Figure 4A). Consistent with these results, APM significantly suppressed MPP^+^-induced secretion of TNFα, IL1β, and IL6 and their mRNA expression in SH-SY5Y cells (Figure S4). 

MPP^+^ causes Ca^2+^-mediated HIF-1α accumulation through the mechanism of ROS production by NOX2 [39]. Therefore, we examined the accumulation of HIFα and NOX2 in response to MPP^+^-induced ROS production. Increased HIFα and NOX2 expression was significantly inhibited in MPP^+^-stimulated SH-SY5Y by APM administration (Figure 4B). 

MPP^+^ causes mitochondrial dysfunction and disturbs Ca^2+^ homeostasis in the ER [38]. APM significantly inhibited MPP^+^-induced GRP78 and CHOP expression in MPP^+^-stimulated SH-SY5Y cells (Figure 4B). Consistent with these results, APM strongly inhibited expression of TNFα, NOX2, and CHOP in MPP^+^-stimulated rat embryo primary mesencephalic neurons (Figure 4C). DCF-DA positive cells were remarkably elevated in MPP^+^-stimulated rat embryo primary mesencephalic neurons (Figure 4D), whereas administration of APM significantly inhibited these levels. These strong expressions were inhibited by APM, which is linked to the outcome of MPP^+^-induced mitochondrial membrane potential disorder observed in Figure 2E. We also confirmed that the MPP^+^-induced TH reduction, αSYN accumulation, and Kca2.2 expression were strongly protected by NAC and 4-PBA (Figure S5a,b). Moreover, the MPP^+^-induced TNFα, NOX2, and CHOP expression were clearly inhibited by BAPTA-AM in SH-SY5Y cells (Figure S3b). These results also suggested that APM could alter MPP^+^-induced neurotoxicity in dopaminergic neuronal cells by inhibiting the Ca^2+^ overload.

### 2.5. APM Ameliorates MPP^+^-Induced Neurotoxicity via ERK/STAT/p65 Signaling Pathway in Dopaminergic Neuronal Cells

**Figure 5:**
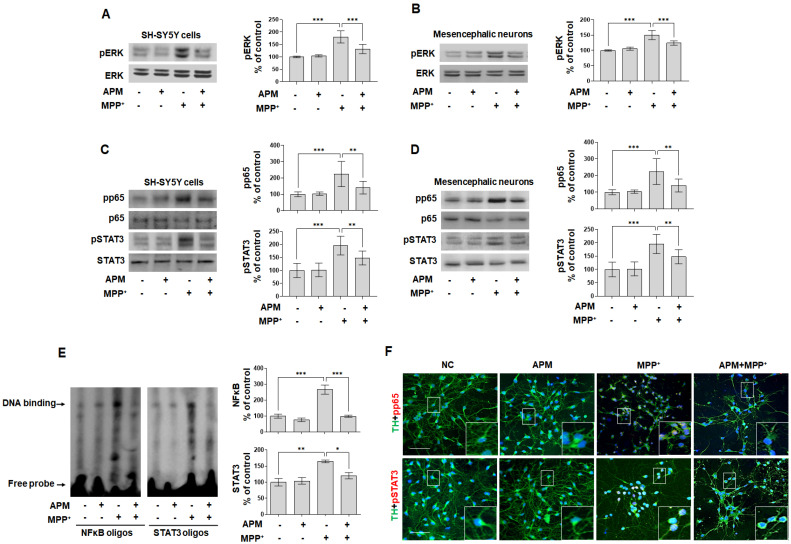
APM strongly inhibited MPP^+^-induced phosphorylation of MAPK-ERK/p65/STAT3 signaling pathway. Dopaminergic neuronal cells were incubated in the presence or absence of APM for 1 h and then treated with MPP^+^ for 12 h. Phosphorylation of ERK in SH-SY5Y cells (**A**) and rat embryo primary mesencephalic neurons (**B**) were analyzed by immunoblotting. MPP^+^-induced NFκB-p65 and STAT3 translocation in SH-SY5Y cells (**C**) and rat embryo primary mesencephalic neurons (**D**) were analyzed by immunoblotting. βActin was used to confirm equal sample loading. (**E**) The DNA-binding activity of NFκB-p65 and STAT3 in nuclear extracts was measured using EMSA. (**F**) Immunofluorescence double staining for TH (green) and pp65 (red) localization, and TH (green) and pSTAT3 (red) localization, in rat embryo primary mesencephalic neurons. Scale bars: 50 μm. Nuclei were stained with DAPI (blue). Immunoblotting and EMSA were quantified by densitometric analysis. The data are representative of three independent experiments and quantified as mean values ± SEM. Tukey’s multiple comparison test, * *p* < 0.05, ** *p* < 0.01, *** *p* < 0.001 compared to normal control.

Dopaminergic neuron loss, oxidative stress, and ER stress induced by MPP^+^ trigger MAPK-ERK/JNK/p38, p65, and STAT potential, disrupting Ca^2+^ homeostasis [12,20]. Accordingly, we investigated whether APM influenced MPP^+^-induced neurotoxicity via regulation of the MAPK-ERK/JNK/p38 signaling pathway. Phosphorylation of MAPK-ERK/JNK/p38 were significantly induced in MPP^+^-stimulated SH-SY5Y cells (Figure 5A and Figure S6a). Interestingly, APM inhibited MPP+-induced pERK-pJNK-pp38, but inhibited pERK most strongly. In addition, it was confirmed that the pERK-MAPK was inhibited by APM in rat embryo primary mesencephalic neurons (Figure 5B). This controlling effect of strong APM on ERK is consistent with our other reports [37,40]. According to these results, since MPP^+^ induced neurotoxicity through the MAPK-ERK signaling pathway, the effects of ERK inhibitor on MPP^+^-induced TH and aSYN expression were investigated. SCH772984 significantly decreased MPP^+^-induced TH reduction and αSYN expression in SH-SY5Y cells, similar to APM (Figure S6b).

Dopaminergic neurotoxins induce cell death and neuroinflammatory responses by NFκB and STAT3 translocation [14,41]. Therefore, we examined the translocation of NFκB and STAT3 in response to MPP^+^-stimulated SH-SY5Y and rat embryo primary mesencephalic neurons. Along with phosphorylation, pp65 and pSTAT3 were translocated from the cytoplasm to the nucleus after MPP^+^ stimulation (Figure 5C,D); however, this was effectively inhibited by APM in dopaminergic neuronal cells. These results were further supported by DNA binding activity. Formation of NFκB-DNA and STAT3-DNA complexes was prominent in nuclear extracts of MPP^+^-stimulated SH-SY5Y cells (Figure 5E). However, formation of these complexes was significantly suppressed in MPP^+^-stimulated SH-SY5Y cells when these cells were treated with APM. We also performed immunofluorescence staining to confirm whether treatment with APM inhibited nuclear translocation of NFκB and STAT3 in MPP^+^-stimulated rat embryo primary mesencephalic neurons. The translocation of pp65 and pSTAT3 expression was observed at the same position as the staining nucleus in MPP^+^-stimulated rat embryo primary mesencephalic neurons (Figure 5F). These expressions were effectively inhibited by APM. These results were consistent with data obtained above. Since MPP^+^-induced neurotoxicity through the NFκB and STAT3 signaling pathway, the effects of each of these inhibitors on MPP^+^-induced TH and αSYN expression were investigated. Bay11-7085 and S3I-201 clearly inhibited MPP^+^-induced TH reduction and αSYN expression in SH-SY5Y cells, similar to APM (Figure S6d). 

To further investigate the interactive role of MPP^+^-induced pERK, pp65, and pSTAT3 translocation, SH-SY5Y cells were treated with Bay11-7085, S3I-201, SCH772984, and APM. MPP^+^-induced pp65 and pSTAT3 translocation was significantly inhibited by SCH772984 and APM (Figure S6c). In addition, MPP^+^-induced pERK was strongly inhibited by Bay11-7085, S3I-201, and APM (Figure S6d). Together, these data confirmed the interaction between MAPK-ERK phosphorylation and p65/STAT3 translocation in dopaminergic neurotoxins, suggesting that APM played an important role in their regulation.

## 3. Discussion

Although the etiology of PD is unclear, evidence suggests that abnormal protein aggregation, mitochondrial dysfunction, and dysregulated Ca^2+^ homeostasis may be involved in neurodegeneration observed in PD [12]. In recent years, changes in Ca^2+^ homeostasis has been suggested to play a key role in the degeneration of dopaminergic neurons [6]. MPP^+^ is shown to selectively increase intracellular Ca^2+^ concentrations in mesencephalic cultures [42]. It was also reported that the increase in cytosolic Ca^2+^ was not caused by MPP^+^-induced oxidative stress but was dependent on Ca^2+^ channel activity and aSYN expression, and these two pathogenic factors acted on PD [43]. A study of potential neuroprotective agents through attenuation of MPP^+^ and Ca^2+^-overload-induced excitotoxicity in SH-SY5Y cells has been reported [44]. Many reports imply that Ca^2+^ is involved in the pathogenesis of PD and, therefore, the regulation of Ca^2+^ might be a potential therapeutic target for neuroprotection in PD [45,46].

It has been suggested that use of APM, a specific selective antagonist of the SK2 channels, has beneficial effects in treating PD [31,34]. We have reported the anti-inflammatory and anti-fibrotic properties of APM in chronic diseases [25,28,29,40]. Recently, we reported that APM suppresses LPS-induced neuroinflammatory responses via SK channels and TLR4 signaling [37]. Our prior studies have investigated the physiological function of APM. However, the molecular mechanisms of MPP^+^-induced neurotoxicity and cellular signaling potential of APM in PD models have not yet been elucidated. The major findings of this study are that APM suppressed TH reduction and αSYN accumulation through the inhibition of MAPK-ERK phosphorylation and pp65/pSTAT3 translocation, and that it ameliorated the neuroinflammatory response and apoptosis in dopaminergic neuronal cells.

Several studies have suggested that reduction of TH, aggregation of αSYN, neuroinflammatory response, and apoptosis of dopaminergic neuronal cells play important roles in both in vitro and in vivo models of PD [6,12,47,48]. Thus, many studies have been conducted to find their inhibitors [7,10,47,49,50,51,52,53]. Furthermore, the membrane-permeable Ca^2+^ chelator, BAPTA-AM, significantly protects cells from oxidative stress, ER stress, and apoptosis [22,54,55,56]. BAPTA-AM and a Ca^2+^ channel blocker suppress αSYN aggregates in HEK293T cells and SHSY-5Y cells treated with KCl54, supporting the notion that dysregulation of cytosolic Ca^2+^ contributes to dopaminergic neurodegeneration [57]. Our result showed that APM inhibited MPP^+^-induced reduction of TH, αSYN expression, neuroinflammatory response, and apoptosis in dopaminergic neuronal cells. APM had an anti-inflammatory and anti-apoptotic effect; this is consistent with our other research reports [25,28,37].

Ca^2+^ signaling plays a vital role in neuronal signaling and altered Ca^2+^ homeostasis in many neuronal diseases including PD [20,44,58]. The rise in intracellular Ca^2+^ rapidly activates CaMKII [59]. Additionally, CaMKII is widely distributed in neurons including mesencephalic neurons [60]. APM inhibited the MPP^+^-induced SK/KCa2.2 and pCaMKII expression in SHSY-5Y cells and rat embryo primary mesencephalic neurons.

CaMKII is a vital regulator of multiple signaling pathways initiated by Ca^2+^ signaling [61]. Activated pCaMKII upregulates downstream ERK, NF-kB, and STAT3 signaling leading to inflammatory response, cell apoptosis, and ultimately neuronal damage [62,63,64]. Recently, several studies reported that the cell signaling control of these transcription factors inhibited neuroinflammation [65,66,67,68,69]. We reported potential candidates for the treatment of neurodegenerative disorders through inhibition of ERK, NFκB, and STAT3 [70]. In accordance with these findings, our present results showed that APM effectively inhibited ERK, NFκB, and STAT3 in MPP^+^-stimulated dopaminergic neuronal cells.

Taken together, APM is believed to be a strong inhibitor of neurotoxicity by regulating mediated to increased SK channels and neurotoxin-mediated signaling pathways in dopaminergic neuronal cells. Thus, APM is a promising candidate for anti-neurotoxic agent, and it can be used for the prevention and treatment of various neurological disorders.

## 4. Materials and Methods

### 4.1. Cell Cultures and Reagents

A dopaminergic human neuroblastoma cell line SH-SY5Y (America Tissue Culture Collection, CRL-2266; ATCC, Manassas, VA, USA), was cultured in a Dulbecco’s Modified Eagle’s Medium (DMEM) medium (Gibco, Grand Island, NY, USA) containing 10% fetal bovine serum (FBS, Gibco) and 1% Anti-Anti (Gibco). Mesencephalic neuron cultures were prepared from the ventral mesencephalic tissues of embryonic day 13–14 rats, as described previously [35,36]. All experimental protocols were approved by the Institutional Animal Care and Use Committee of the Daegu Catholic University Medical Center (EXP-IRB number: DCIAFCR-191112-07-Y) in accordance with criteria outlined in the Institutional Guidelines for Animal Research. Briefly, dissociated cells were seeded at 1 × 10^5^/well to poly-d-lysine and laminin-coated 24-well plates. Cells were cultured in a Dulbecco’s modified Eagle’s medium/Ham’s F-12 medium (Gibco) containing ITS premix (Sigma-Aldrich, St Louis, MO, USA) and 1% penicillin-streptomycin (Gibco). Cell cultures were maintained at 37 °C in a humidified atmosphere of 5% CO_2_.

The sources of the following reagents were: MPP^+^ (Sigma-Aldrich); an intracellular Ca^2+^ chelator, BAPTA-AM (Sigma-Aldrich); a specific inhibitor for ROS, NAC (Cell Signaling, Danvers, MA, USA); a specific inhibitor for ER stress, phenyl butyric acid, 4-PBA (Sigma-Aldrich); a specific for ERK, SCH772984 (Cell Signaling); a specific for NF-κB, Bay11-7085 (Sigma-Aldrich); a specific for STAT3, S3I-201 (Sigma-Aldrich); anti-Kca2.2 (Millipore, Bedford, MA, USA); anti-pCaMKII and anti-CaMKII (Novus, Littleton, CO, USA); anti-TNFα, anti-IL1β, and anti-IL6 (Abcam, Cambridge, MA, USA); anti-HIF1α, anti-Bcl-xL, and anti-Bax (Santa Cruz Biotechnology, Dallas, TX, USA); anti-NOX2 (Thermo Fisher Scientific, Waltham, MA, USA); anti-TH, anti-αSYN, anti-Caspase3, anti-PARP, anti-GRP78, anti-CHOP, anti-pERK, anti-ERK, anti-pJNK, anti-JNK, anti-pp38, anti-p38, anti-pp65, anti-p65, anti-pSTAT3, anti-STAT3, and anti-β-actin, and horseradish peroxidase-conjugated secondary antibodies (Cell Signaling). Immunoblots were detected using an enhanced chemiluminescence reagent (Amersham Bioscience, Amersham, UK).

### 4.2. Morphology Examination

Morphological changes in cells were observed under an inverted phase-contrast microscope (Olympus CKX41SF, Tokyo, Japan). The effect of APM on MPP^+^-induced neurotoxicity was observed for 24 h. The photographs were taken at ×200 or ×400 magnification using a digital camera.

### 4.3. Cytotoxicity Assay

To evaluate the effect of APM on MPP^+^-stimulated proliferation of SH-SY5Y cells, cells were plated in 96-well culture plates at 1 × 10^5^ cells/mL in culture medium and allowed to attach for 24 h. Media were then discarded and replaced with new medium containing various concentrations of MPP^+^ and APM. Cell viability was analyzed using the Cell Counting Kit (CCK8; Dojindo Laboratories, Kumamoto, Japan) assay according to the manufacturer’s instructions. The absorbance at 450 nm was assessed using a microplate reader (Thermo Fisher Scientific).

### 4.4. Quantitative Real-Time Polymerase Chain Reaction (PCR) Analysis

mRNA transcription of cytokines was analyzed by qRT-PCR. Total RNA was extracted from SH-SY5Y cells using TRIzol Reagent (Thermo Fisher Scientific) according to the manufacturer’s recommendations. Reverse transcription reaction was performed using EcoDry Premix Kit (TaKaRa, Tokyo, Japan). cDNA was subjected to qRT-PCR using SYBR Green Mix kit (Toyobo, Osaka, Japan) and the CFX Connect real-time PCR system (Bio-Rad Laboratories, Hercules, CA, USA). Primers, synthesized at Microgen (Daejon, Korea), were as follows: for TNF-α, 5′-TCT CGA ACC CCG AGT GAC AA-3′ (sense) and 5′-TGA AGA GGA CCT GGG AGT AG-3′ (antisense); for IL-6, 5′-CAC AGA CAG CCA CTC ACC TC-3′ (sense) and 5′-TTT TCT GCC AGT GCC TCT TT-3′ (antisense); for β-actin, 5′-CTT CCT GGG CAT GGA GTC CT-3′ (sense) and 5′-GGA GCA ATG ATC TTG ATC TT-3’ (antisense). β-actin served as a normalization control. The relative RNA expression of each gene was analyzed using the 2^−ΔΔ*C*T^ method as previously reported [37]. 

### 4.5. Enzyme-Linked Immunosorbent Assay (ELISA)

The culture medium of the cells was harvested, and cytokine production (TNFα, IL1β, and IL6) in the supernatant was measured with a solid phase sandwich enzyme-linked immunosorbent assay (ELISA) using a Quantikine TNFα, IL1β, and IL6 kit (R&D systems, Minneapolis, MN, USA) according to the manufacturer’s instructions.

### 4.6. Immunoblot Analysis

Cytosolic and nuclei protein fractions were obtained as described [34]. Protein samples were prepared from the cultured SH-SY5Y cells and rat embryo primary mesencephalic neurons with a protein extraction buffer (Cell Lytic™ M; Sigma) and NE-PER Nuclear and Cytoplasmic Extraction Kit (Thermo Fisher Scientific). The protein concentration of the samples was measured with Bio-Rad Bradford kit (Bio-Rad Laboratories). The protein samples were loaded on precast gradient polyacrylamide gels (Bolt™ 4–12% Bis-Tris Plus Gels; Thermo Fisher Scientific) and transferred to nitrocellulose membranes (GE Healthcare, Madison, WI, USA) by using Bolt™ Mini Blot Module and Mini Gel Tank (Thermo Fisher Scientific), according to the manufacturer’s recommendations. The membrane blocked with 5% bovine serum albumin was probed with a primary antibody and horseradish peroxidase-conjugated secondary antibody. The chemiluminescent substrate (Thermo Fisher Scientific) was used to detect the protein bands. Image analyses were performed using the ChemiDoc™ XRS+ (Bio-Rad Laboratories). 

### 4.7. Terminal Deoxynucleotidyl-Transferase-Mediated dUTP Nick End Labelling (TUNEL) Staining

Apoptotic cells were detected in situ by TUNEL assay using an In Situ Cell Death Detection kit (Roche Applied Science, Penzberg, Germany). Following this, the cells were resuspended in permeabilization solution for 2 min on ice. Cells were washed by PBS three times, resuspended in TUNEL reaction buffer mixture, and incubated in the dark at 37 °C for 1 h. Cells were counterstained with DAPI (excitation/emission = 330–380 nm/460 nm, ImmunoChemistry, Bloomington, MN, USA). Immunolabeling was examined by an Eclipse Ti-U confocal microscope and processed with NIS-Elements C ver. 4.20 software (Nikon, Tokyo, Japan). A total of 10 fields-of-view were randomly selected for analysis. 

### 4.8. JC-1 Mitochondrial Transmembrane Potential Assay

To measure the mitochondrial transmembrane potential, JC-1 dye (Sigma-Aldrich), a sensitive fluorescent probe, was used. Fluorescence microscopy with a 488 nm filter was used for the excitation of JC-1. Emission filters of 535 and 595 nm were used to quantify the population of mitochondria with green (JC-1 monomers) and red (JC-1 aggregates) fluorescence, respectively. Immunolabeling was examined by an Eclipse Ti-U microscope (Nikon). A total of 10 fields-of-view were randomly selected for analysis.

### 4.9. Detection of Intracellular Ca^2+^ and ROS Expression

For evaluating the intracellular Ca^2+^ level and oxidative stress levels in rat embryo primary mesencephalic neurons, FluoForte Calcium Assay Kit (Enzo Life Sciences, Ann Arbor, MI, USA) and 2′,7′-dichlorofluorescin diacetate (DCFDA)/H2DCFDA-Cellular ROS Assay Kit (Abcam) were used. Cells were stained according to each manufacturer’s recommended protocol. Cells were counterstained with DAPI (excitation/emission = 330–380 nm/460 nm, ImmunoChemistry). The fluorescence signal was detected and observed using an Eclipse Ti-U and confocal microscope (Nikon). A total of 10 fields-of-view were randomly selected for analysis.

### 4.10. Immunofluorescent Staining

Cells were incubated with primary antibodies for 1 h at room temperature. After washing, they were incubated with the Alexa Flour 488 (excitation/emission = 495/519 nm, green, Invitrogen, Carlsbad, CA, USA) and Alexa Flour 594 (excitation/emission = 590/617 nm, red, Invitrogen) for 30 min at room temperature. Cells were counterstained with DAPI (excitation/emission = 330–380 nm/460 nm, ImmunoChemistry). Slides were mounted using ProLong^®^ Gold antifade reagent (Molecular Probes^®^ by Life Technologies^TM^, Carlsbad, CA, USA). Immunolabeling was examined using an Eclipse Ti-U and confocal microscope (Nikon).

### 4.11. Electrophoretic Mobility Shift Analysis

Nuclear extract fractionation from dopaminergic neuronal cells was conducted using an NE-PER Nuclear and Cytoplasmic Extraction Kit (Thermo Fisher Scientific) according to the manufacturer’s instructions. The lightshift chemiluminescent electrophoretic mobility shift analysis (EMSA) Kit (Thermo Fisher Scientific) was used for the EMSA to analyze the expression of NFκB and STAT3. The consensus NFκB binding site (5′-AGT TGA GGG GAC TTT CCC AGG C-3′) and STAT3 binding site (5′-CTT CAT TTC CCG TAA ATC CCT AAA GCT-3′) were used as DNA binding oligos.

### 4.12. Statistical Analysis

All data analysis was performed with the GraphPad Prism 9 (GraphPad Software, Inc., San Diego, CA, USA) using either a one-way ANOVA with Tukey’s post hoc test for multiple comparisons and data are presented as the mean ± SEM (* *p* < 0.05, ** *p* < 0.01, *** *p* < 0.001).

## Data Availability

Not applicable.

## Supplementary Materials

The following supporting information can be downloaded at: https://www.mdpi.com/article/10.3390/ijms232315255/s1.
