# Supplementary material for: Effects of Apamin on MPP+-Induced Calcium Overload and Neurotoxicity by Targeting CaMKII/ERK/p65/STAT3 Signaling Pathways in Dopaminergic Neuronal Cells
Source: Int J Mol Sci. 2022 Dec 3;23(23):15255. doi: 10.3390/ijms232315255 (PMC9736188; doi:10.3390/ijms232315255)

Effects of apamin on MPP+-induced calcium overload and neurotoxicity by targeting CaMKII/ERK/p65/STAT3 signaling pathways in dopaminergic neuronal cells

Figure S1: Effects of APM and MPP^+ on viability and morphology of SH-SY5Y cells. SH-SY5Y cells were incubated in the presence or absence of APM for 1 h and then treated with MPP^+ for 24 h. (a) SH-SY5Y cells were treated with MPP^+ (1– to 5 mM) for 24 h and CCK assays were conducted. (b) Cell morphology changes, Magnifications ×200. (c) Changes in TH, aSYN, and Kca2.2 expression according to the concentration of MPP^+. (d) SH-SY5Y cells were treated with APM (0.1 to 2 μg/ml) for 24 h and CCK assays were conducted. (e) APM significantly increased the viability of MPP^+-stimulated SH-SY5Y cells. βActin was used to confirm equal sample loading. Immunoblotting was quantified by densitometric analysis. The data are representative of three independent experiments and quantified as mean values ± S.E or SEM (one-way ANOVA and Tukey's multiple comparison test; * $p < 0.05$, ** $p < 0.01$, *** $p < 0.001$ compared to normal control)

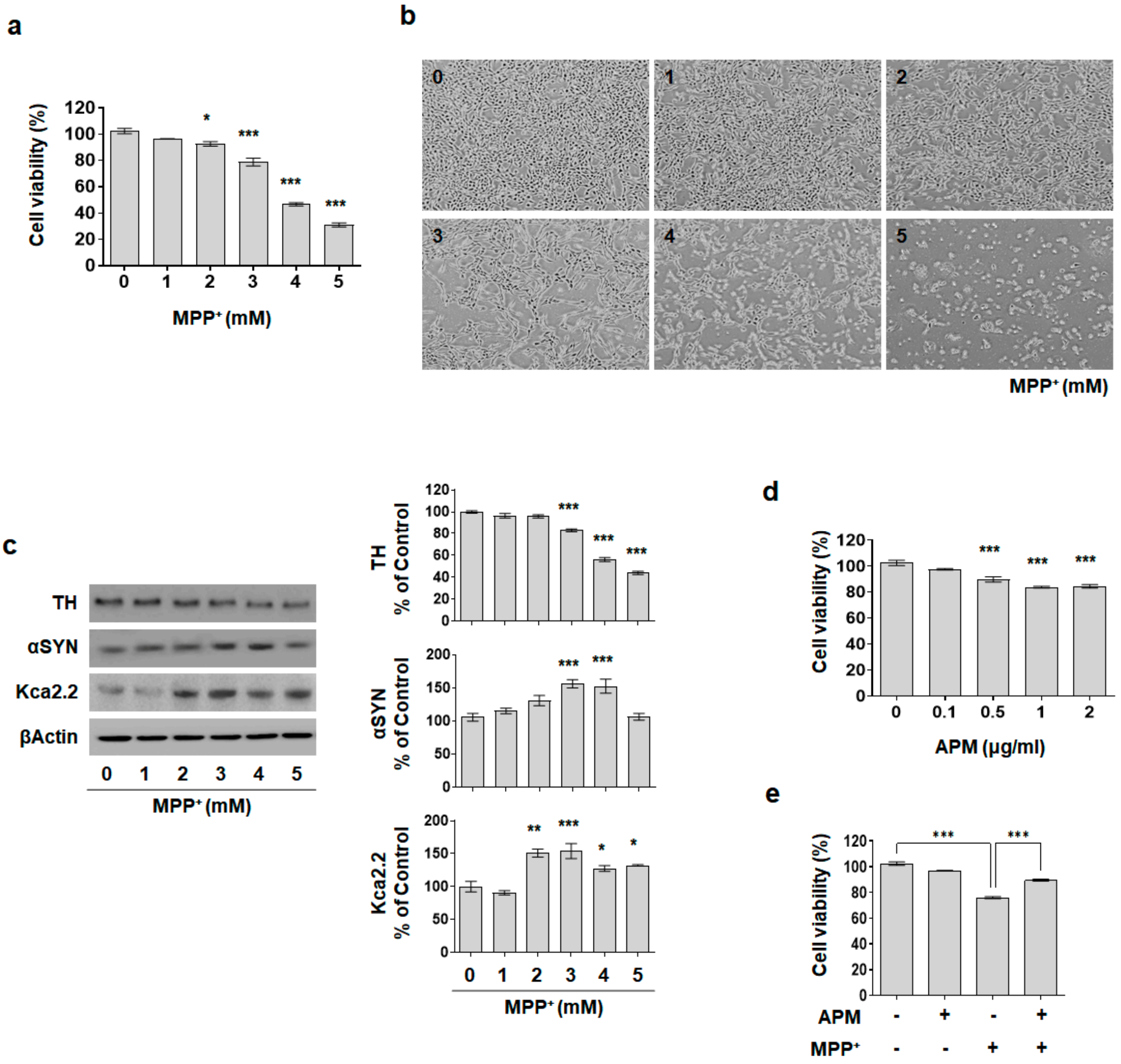

Figure S2: Effects of APM and MPP^+ on viability and morphology of rat embryonic primary mesencephalic neurons. Rat embryonic primary mesencephalic neurons were incubated in the presence or absence of APM for 1 h and then treated with MPP^+ for 24 h. (a) Rat embryonic primary mesencephalic neurons were treated with MPP^+ (10-200 μM) for 24 h and CCK assays were conducted. (b) Cell morphology changes, Magnifications ×200. (c) Changes in TH, aSYN, and Kca2.2 expression according to the concentration of MPP^+. (d) Rat embryonic primary mesencephalic neurons were treated with APM (0.1 to 2 μg/ml) for 24 h and CCK assays were conducted. (e) APM significantly increased the viability of MPP^+-stimulated rat embryonic primary mesencephalic neurons. βActin was used to confirm equal sample loading. Immunoblotting was quantified by densitometric analysis. The data are representative of three independent experiments and quantified as mean values ± S.E or SEM (one-way ANOVA and Tukey's multiple comparison test; * $p < 0.05$, ** $p < 0.01$, *** $p < 0.001$ compared to normal control)

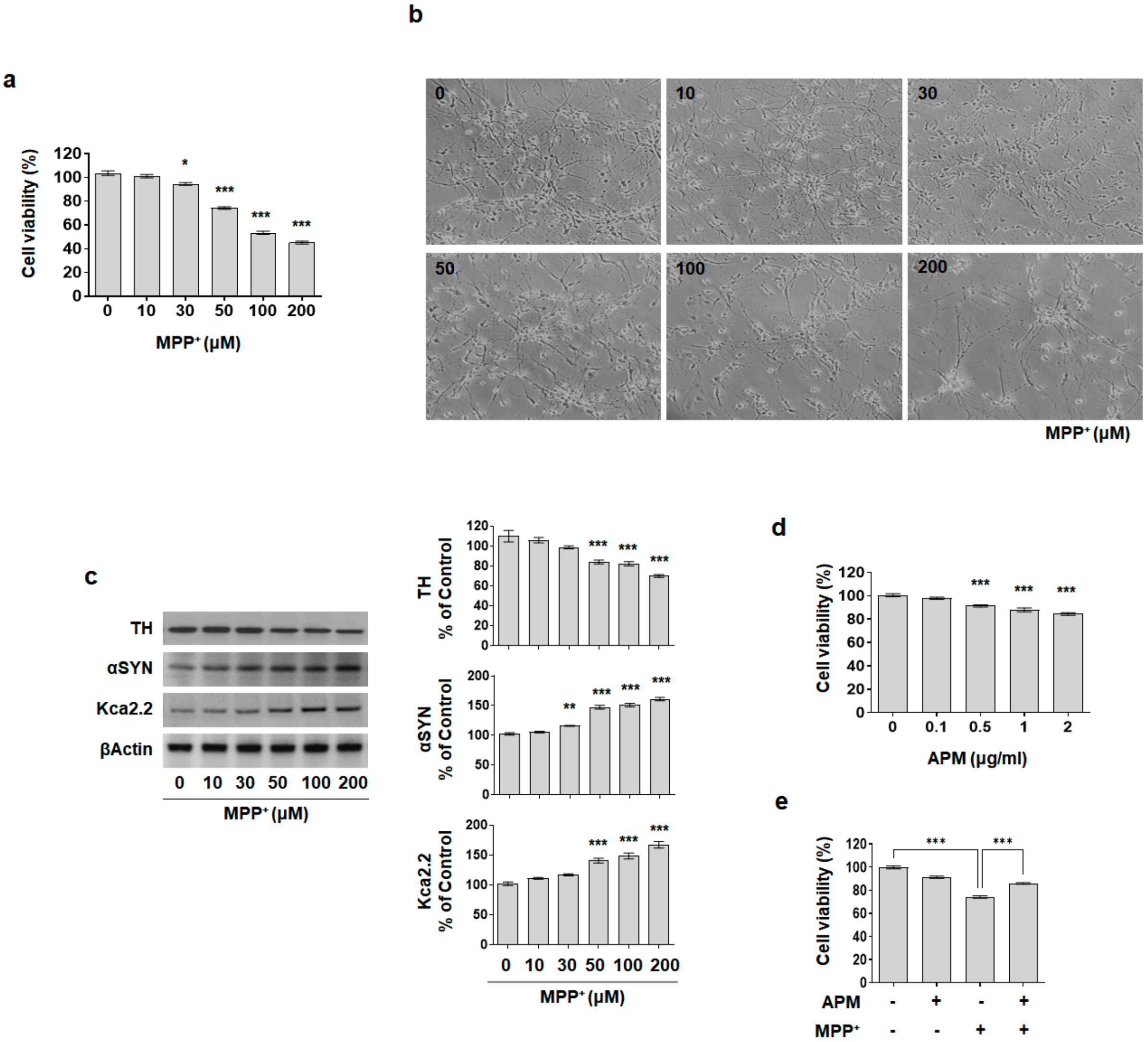

Figure S3: Effects of Ca^{2+} chelator, BAPTA-AM on MPP^+-stimulated SH-SY5Y cells. SH-SY5Y cells were treated with APM and BAPTA-AM (5 μM) for 1 h and then treated with MPP^+ for 24 h. Expression of TH reduction and aSYN (a), TNFα, NOX2 and CHOP (b) were significantly inhibited in MPP^+-stimulated SH-SY5Y cells by APM or BAPTA-AM. βActin was used to confirm equal sample loading. Immunoblotting was quantified by densitometric analysis. The data are representative of three independent experiments and quantified as mean values ± SEM. Tukey's multiple comparison test, ** $p < 0.01$, *** $p < 0.001$ compared to normal control.

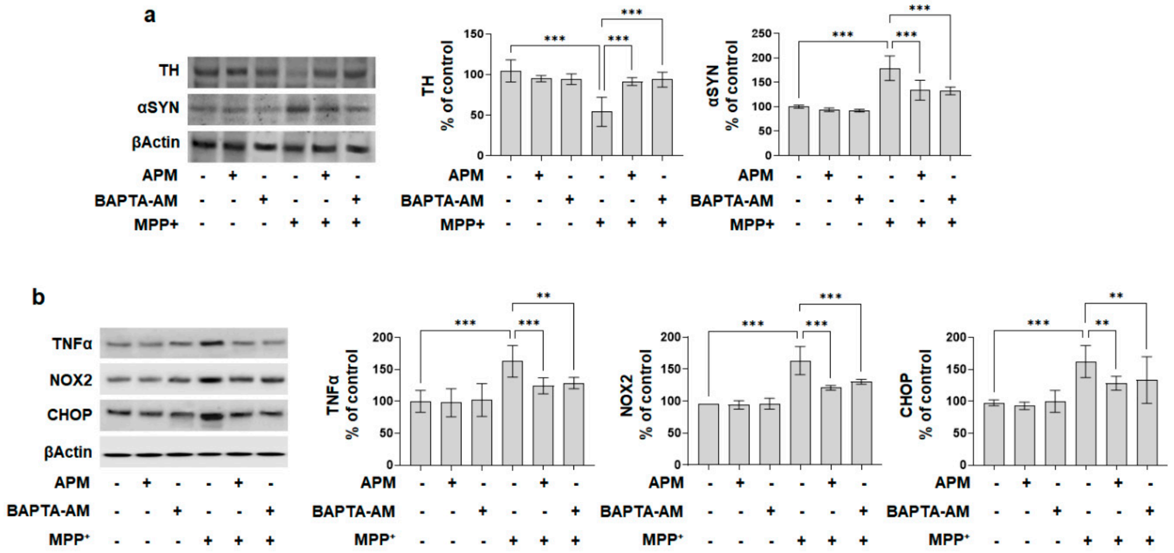

Figure S4: APM alleviated MPP^+-induced neuroinflammatory response. SH-SY5Y cells were treated with APM for 1 h and then treated with MPP^+ for 24 h. Production of TNFα, IL1β and IL6 were analyzed by ELISA (a) and real-time PCR (b) in SH-SY5Y cells. The data are representative of three independent experiments and quantified as mean values ± SEM. Tukey's multiple comparison test, * $p < 0.05$, ** $p < 0.01$, *** $p < 0.001$ compared to normal control.

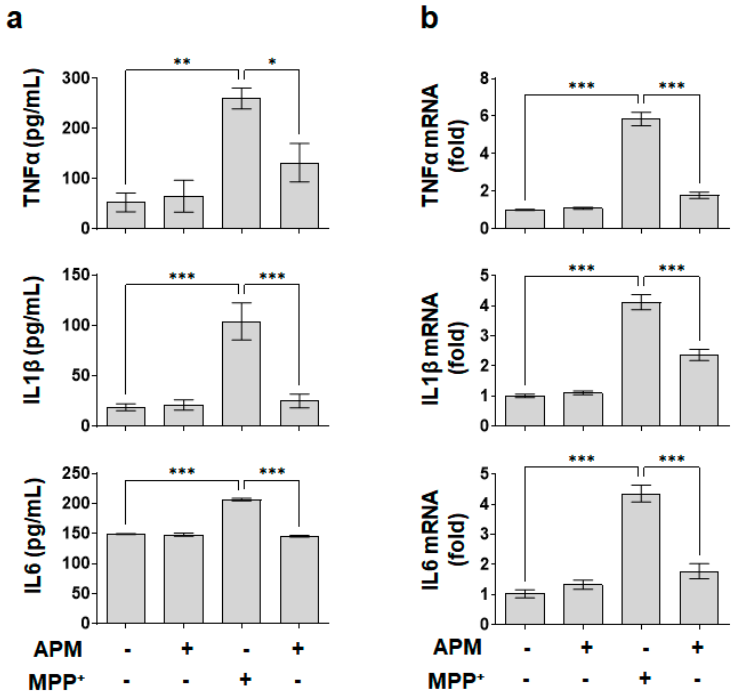

Figure S5: Effects of ROS and ER stress on MPP^+-induced TH reduction, aSYN and Kca2.2 expression. SH-SY5Y cells were treated with APM, an ROS inhibitor, NAC 5 μM and ER stress inhibitor, 4-PBA, 5 μM for 1 h and then treated with MPP^+ for 24 h. Expression of TH reduction, aSYN and Kca2.2 were significantly inhibited in MPP^+-stimulated SH-SY5Y cells by NAC (A) and 4-PBA (b). βActin was used to confirm equal sample loading. Immunoblotting was quantified by densitometric analysis. The data are representative of three independent experiments and quantified as mean values ± SEM. Tukey's multiple comparison test, * $p < 0.05$, ** $p < 0.01$, *** $p < 0.001$ compared to normal control.

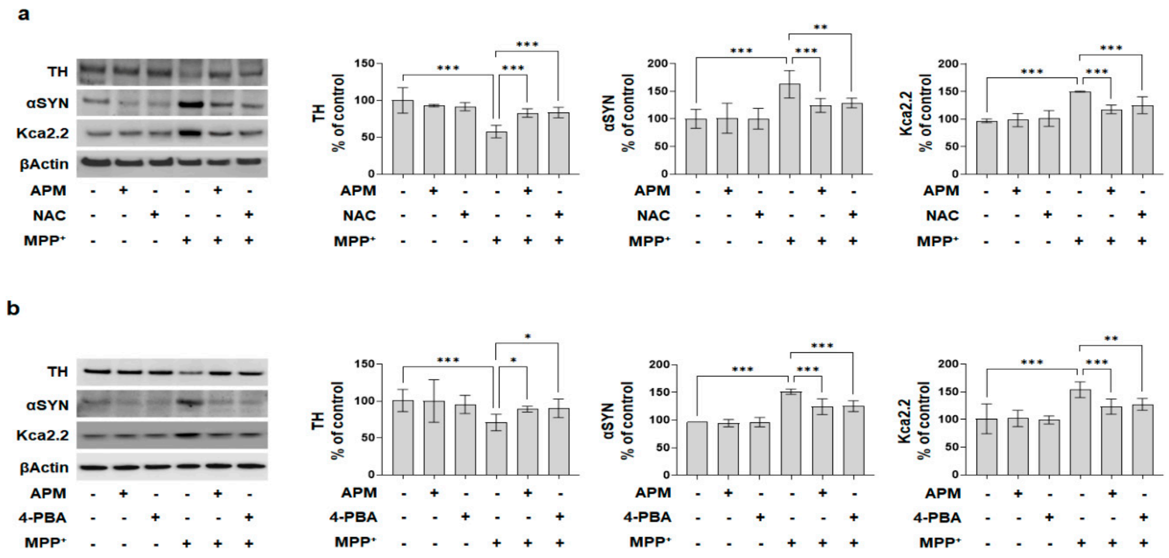

Figure S6: APM inhibits the interaction of MAPK-ERK with p65/STAT3 in MPP+-induced neurotoxicity. SH-SY5Y cells were treated with an ERK inhibitor (SCH772984, 10 μM), a NFκB inhibitor (Bay11-7085, 10 μM), STAT3 (S3I-201, 10 μM) and APM for 1 h and then treated with MPP+ 12 h and 24 h. Expression of pp65 and pSTAT3 in nuclear lysate and expression of p65 and STAT3 in cytoplasmic lysate were conducted by immunoblotting. (a) Effects of APM on MPP+-induced MAPK-JNK/p38 phosphorylation. MPP+-induced TH reduction and αSYN expression (b), and pp65/pSTAT3 translocation (c) were inhibited when the ERK signal pathway of these cells were downregulated. (d) Bay11-7085 and S3I-201 significantly inhibits MPP+ induced TH reduction, αSYN and pERK. βActin was used to confirm equal sample loading. Immunoblotting was quantified by densitometric analysis. The data are representative of three independent experiments and quantified as mean values ± SEM. Tukey's multiple comparison test, * $p < 0.05$, ** $p < 0.01$, *** $p < 0.001$ compared to normal control.

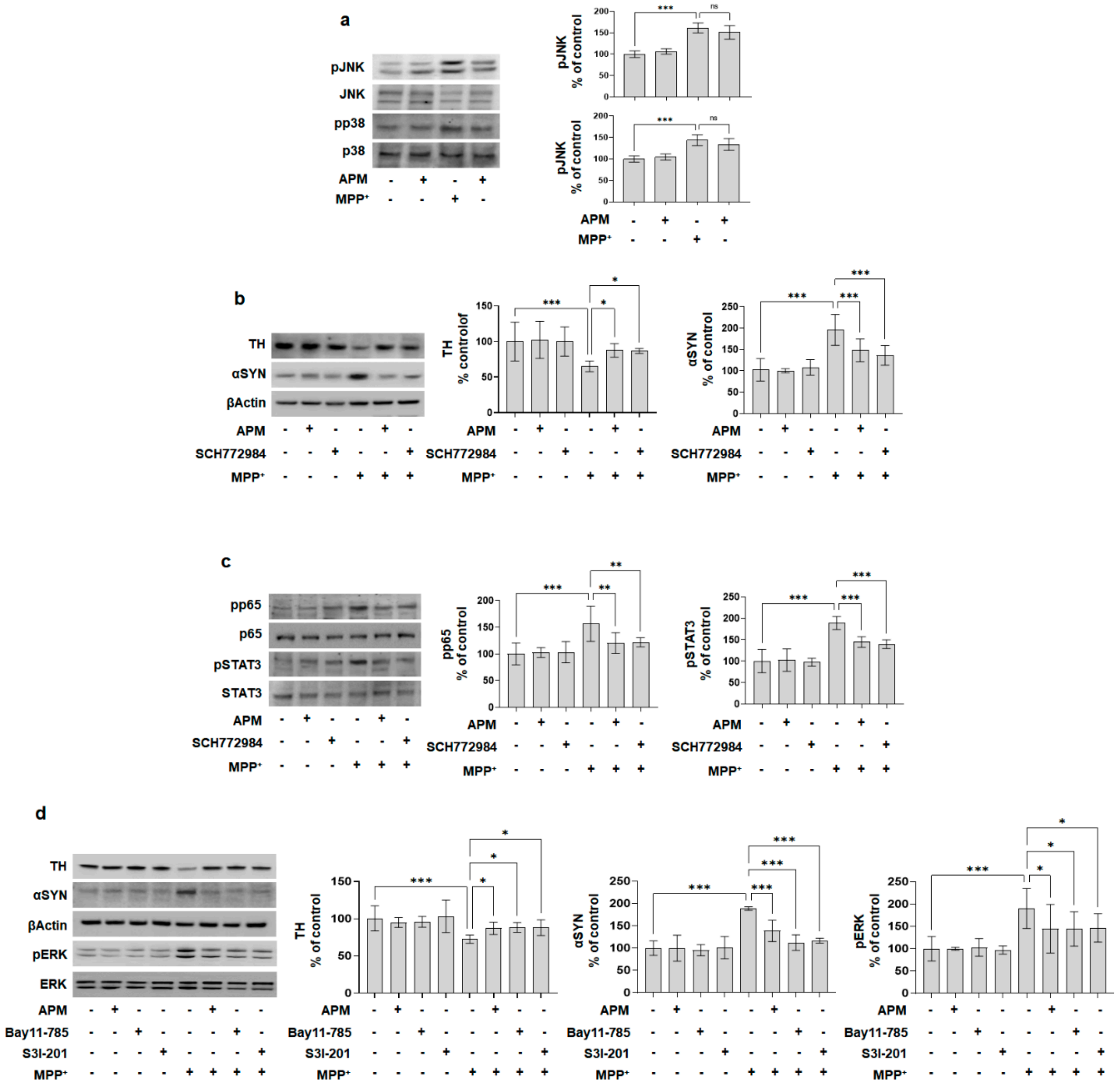

Supplement: Supplementary file 1 [file ijms-23-15255-s001.zip › ijms-2013404-supplementary.pdf]
